# Supercritical fluid extraction and encapsulation of Rivas (*Rheum ribes*) flower: Principal component analysis (PCA)

**DOI:** 10.1016/j.heliyon.2025.e41746

**Published:** 2025-01-06

**Authors:** Seyyed Ali Hoseini, Mohsen Vazifedoost, Bahareh Hajirostamloo, Zohreh Didar, Mohamad Mehdi Nematshahi

**Affiliations:** a Department of Food Science and Technology, Neyshabur Branch, Islamic Azad University, Neyshabur, Iran; b Department of Food Science and Engineering, Hamedan Branch, Islamic Azad University, Hamedan, Iran

**Keywords:** Rivas, Ethanol-modified supercritical CO_2_, Bioactivity, GC-MS, Encapsulation, Morphology, PCA

## Abstract

Supercritical CO_2_ modified by polar solvents can extract a wide variety of polar and non-polar chemical components compared to conventional methods. The current study aims to extract Rivas (Rheum ribes) flower using the ethanol modified supercritical CO_2_ (SCO_2_-EOH) method; analyze its chemical compounds and bioactivity, encapsulate the extract in maltodextrin, gum-Arabic (GA), and their combination (GA + MD) using the spray drying method and investigate the differences among microparticles using Principal Component Analysis (PCA). The Rivas extract obtained by the SCO_2_-EOH method was a rich source of unsaturated fatty acids (mainly linoleic acid: 57.58 %), phytosterols (mainly sitosterol: 197.02 and campesterol: 144.47 mg/100g), terpenoids (mainly camphor: 17.52 %; and 1,8-cineol: 10.91 %) and phenolics (mainly m-coumaric acid: 48.22; luteolin: 38.07 and gallic acid: 26.25 mg/g). The yield of Rivas extract was 1.62 ± 0.27 %. The extract bioactivity was as follows: antioxidant activity of 89.6 ± 1.39 %; total phenolic content of 306.19 ± 13.59 mg GAE/g; total flavonoid content of179.84 ± 5.77 mg QE/g and a comparable antimicrobial effect to synthetic antimicrobials against *E. coli*, *L. monocytogenes*, and *A. fumigatus*. The encapsulation efficiency of microparticles was 90.53 % for MD to 93.23 % for GA + MD (P < 0.05). The microparticles had irregular semi-spherical shapes with wrinkled surfaces. According to the PCA, MD showed the best solubility and the lowest price, making it a cost-effective ingredient to improve the nutritional-value of food formulations. If the stability of bioactive compounds is more important, GA + MD will be the best choice.

## 1 Introduction

Rhubarb (Rheum ribes) is a native plant of Iran, known locally as “Rivas” and belongs to the Polygonaceae family. It is primarily found in Western and North-western Iran, Northern Iraq, and Lebanon [1]. Rheum species are a valuable source of various phytochemical compositions including flavonoids, tannins, phenolic compounds, vitamins, elements, organic acids and anthraquinones [2]. Additionally, the hexane extract obtained from rhubarb flowers is reported to be a good source of unsaturated fatty acids and some hydrocarbons [3].

Due to the presence of various valuable biochemical compounds in R. ribes, researchers are interested in the extraction process from the plant**.** The extract could be a suitable alternative to chemical preservatives in the food industry due to its antimicrobial and antioxidant potential [4]. Moreover, natural plant extracts are widely utilized in the pharmaceutical industry as a source of various bioactive compounds with antiaging, anticancer, antioxidant, anti-inflammatory, antidiabetic and antimicrobial potential [5].

Generally, the extraction process of these compounds relies on chemical solvents such as hexane, diethyl ether and methanol. These solvents are non-renewable, toxic, and pollute the environment [6,7]. Supercritical fluids offer a green and effective approach to extracting bio-chemicals, particularly complex organic compounds, from plants. This extraction method combines liquid solvating abilities with gaseous transport characteristics [8]. Among supercritical fluids, carbon dioxide (CO_2_) is the most common due to its abundance, inert and non-toxic nature, ease of recovery and low critical temperature and pressure [9]. Previous studies have examined the impact of the SCO_2_ extraction method on the chemical composition and bioactivity (e.g., antimicrobial and antioxidant potential) of various extracts including soybean residue [6], *Ruta chalepensis* [10] and black rosehip [7]. However, CO_2_ has a nonpolar nature, resulting in low extraction efficiency for highly polar functional groups such as –OH and –COOH [11]. By adding co-solvents or modifiers with a polar nature like alcohol, water, and acids to CO_2_, the extraction efficiency of phenolic compounds can be enhanced. This enhancement can be attributed to chemical and/or physical interactions between the solute and solvent, swelling of the solid matrix, and improved solute transport [12]. Therefore, the potential of using ethanol as a co-solvent to improve the recovery and solubility of flavonoid and phenolic compounds of various plants such as *Arachis hypogea* [13], Juglans nigra, black walnut, husks [8], *Celastrus hindsii* leaf [14], rice husk [15], Rambutan Seed Waste [16] and chokeberry pomace [17] in SCO_2_ has been studied. It has been reported that extracts obtained from the SCO_2_-EOH method contain a wider variety of polar and non-polar chemical components compared to conventional plant essential oils or extracts [18,19]. However, the extraction of Rivas flower using this method has not been studied. Therefore, the results of the current study could provide valuable data.

Since bioactive compounds are sensitive to environmental and process stresses, encapsulation is a promising solution to maintain these compounds [20,21]. Among various encapsulation techniques, spray drying is known as a flexible, cost-effective, and industrial method [22], that has been successfully applied to encapsulate various bioactive compounds [[23], [24], [25], [26]]. On the other hand, maltodextrin and gum Arabic are widely considered as two of the most common materials used for encapsulation. Their popularity can be related to several factors, including their safety, high water solubility, excellent barrier properties, good biocompatibility, neural taste, odor and color as well as their appropriate viscosity, and emulsifying properties. These wall materials have shown great potential for the encapsulation of various bioactive compounds such as blackberry [25], saffron [27], date fruit [28] and *Arctium lappa* L. root [29].

To the best of the authors knowledge, no published research has addressed the chemical composition, antioxidant and antimicrobial potential of R. ribes extracted based on SCO_2_-EOH. Therefore, in the current study, the extract of Rivas flower was obtained using the SCO_2_-EOH method. The free fatty acids profile, phytosterol, terpenoid and phenolic compounds of the extract were determined using GC-MS and HPLC methods. The antimicrobial and antioxidant potentials of Rivas flower extract were investigated. The extract was encapsulated into maltodextrin and/or gum Arabic as cost-effective carrier agents. The differences between the physicochemical properties of various microparticles were evaluated using Principal Component Analysis (PCA) to choose the most suitable microparticle.

## 2 Materials and methods

### 2.1 Materials

One kilogram of dried Rivas flower (Rheum ribes L.) was purchased from a local market (Neyshabur, Khorasan Razavi, Iran). The dried sample was ground (KRUPS.GVX231 Expert Burr Grinder, Mexico) and passed through a sieve with a mesh size of 40. All chemicals and reagents used in the analysis were of analytical grade and obtained from Sigma (Sigma Chemicals Co, St. Louis, Missouri, USA), Dr. Mojalali Chemical Complex Co. (Tehran, Iran) and Merck (Darmstadt, Germany). The microbial strain cultures including *Escherichia coli* ATCC 25922; *Listeria monocytogenes* PTCC: 1783; *Aspergillus fumigatus* PTCC5009) were acquired from the Iranian Research Organization for Science and Technology (IROST; Karaj, Iran).

### 2.2 Extraction process

**Fig. 1:**
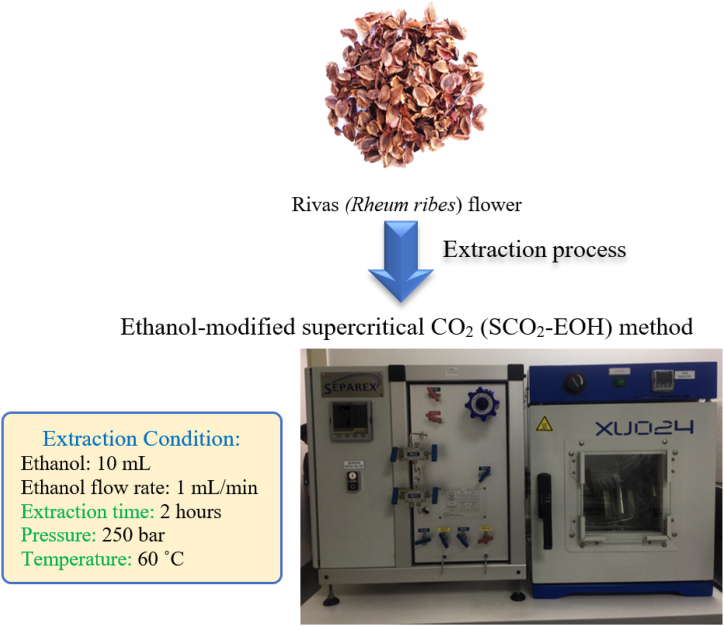
Diagram of the supercritical extraction setup.

Three grams of Rivas flower powder were transferred into a 50-mL tube with glass beads. The extraction process was carried out using a supercritical CO_2_ extraction system (Separex 4325, Separex Co., Champigneulles, France). Liquefied CO_2_ (−5 °C, 1 mL/min) was introduced into the extraction system along with 10 mL ethanol at a flow rate of 1 mL/min using an HPLC pump [14]. A Box-Behnken design with 15 runs at three levels (−1, 0, and 1) was utilized to optimize the extraction parameters. The independent variables included extraction time (1, 2, and 3 h), pressure (200, 300, and 400 bar), and temperature (40, 60 and 80 °C). The parameters were optimized to achieve the highest yield (1.62 ± 0.27 g/100 g dry weight) and antioxidant activity against DPPH radicals (89.6 ± 1.39 %). The optimized conditions were as follows: extraction time of 2 h; pressure of 250 bar and temperature of 60 °C (Fig. 1). The antioxidant activity.

### 2.3 Chromatography-mass Spectrometry (GC-MS)

The Volatile fraction of R. ribes extract including fatty acids, phytosterols and terpenoids was evaluated using a Gas Chromatography-Mass Spectrometry (GC-MS) system (Agilent 7890 Agilent Technologies, USA). The conditions of the instrument were as follows: Injection volume: 1 μL; oven temperature: 40–300 °C (maintained for 2 min at the initial temperature; 40–150 °C at 5 °C/min, 150 °C −300 at 15 °C/min); Injection concentration: 5 mg/mL; injector temperature 280 °C; ion source temperature: 200 **°**C; Carrier gas: Helium (7 psi) at a flow rate of 2 mL/min; Electron ionization 70 eV. Identification of the extract components was done based on computer matching with the WILEY-MS library, literature studies, and standards. The system was controlled by means of Agilent MSD Chemstation software [30,31].

### 2.4 High-performance liquid chromatography (HPLC)

The phenolics of Rivas flower extract were analyzed using HPLC (Agilent Technologies 1200 series، Germany) equipped with a UV detector and a C18 reverse phase column (5 μm ID, 4.6 × 150 mm FT; Zorbax eclipse (XDB), at a temperature of 30 °C). The analysis conditions were as follows: a run time of 40 min; a flow rate of 1 mL/min; a wave length of 280 nm and an injection volume of 20 μL. The Chemstation software (Waldbronn, Germany) was utilized to control the device and acquire data [32].

### 2.5 Total phenolic content (TPC)

The total phenolic content (TPC) of the extract was evaluated using the Folin & Ciocalteu method. Specifically, 1 mL of the extract was mixed with 1 mL of Folin-Ciocalteu reagent and shaken for 5 min. Then, 10 mL of Na_2_CO_3_ solution was added to the mixture, bringing the total volume to 25 mL with distilled water. The solution was stored in a dark place for 60 and the absorbance was read at 750 nm against the control sample (distilled water) using a spectrophotometer (Shimadzu UV-2501, Tokyo, Japan). The results were expressed as milligrams of gallic acid equivalent per gram of dry weight of extract (mg GA/g) [33].

### 2.6 Total flavonoid content (TFC)

The TFC of Rivas extract was evaluated using the aluminum chloride colorimetric method. Briefly, 1 mL of the extract was mixed with 5 mL of distilled water and 0.3 mL of a 5 % NaNO_2_ solution. After 5 min, 0.3 mL of a 10 % AlCl_3_.H_2_O solution was added. Following an additional 6 min, 2 mL of 1 M sodium hydroxide (NaOH) was introduced to the mixture and the volume was reached to 25 mL with deionized water. The absorbance of the solution was measured at a wavelength of 510 nm using a spectrophotometer [34]. The total flavonoid content was expressed as milligrams of Quercetin equivalents per gram of dried extract (mg QE/g).

### 2.7 Antioxidant activity

The antioxidant activity of Rivas extract was measured spectrophotometrically based on DPPH֯ (1,1-diphenyl-2-picrylhydrazyl) free radical scavenging capacity. The extract (0.2 mL) was mixed with 2 mL of a 0.4 mM DPPH methanolic solution and 9 mL of Tris- HCl. Then incubated for 90 min at room temperature in a dark place. The absorption of the extract (A_sample_) was read at a wavelength of 515 nm against a blank sample (A_blank_: sample without extract).

The DPPH free radical scavenging capacity (RSC %) was calculated using equation (1):

(Eq.1) $$RSC\;\left(\%\right)=\frac{A_{blank}-A_{sample}}{A_{blank}}\times 100$$

### 2.8 Antimicrobial effect

The antimicrobial effect of the Rivas extract was evaluated using the paper disc diffusion method [35,36] with slight modifications. Mueller-Hinton agar (for *E.coli and L. monocytogenes*) was used for bacteria and Sabouraud Dextrose agar was used for fungi (*A. fumigatus*). A microbial suspension (0.1 mL) was spread on the agar surface in Petri dishes. Paper discs with a diameter of 6 mm were soaked in Rivas extract concentration of 50–400 μg/mL, then dried at 37 °C for 12 h. These dried discs were placed on the plates which were then incubated at 37 °C for 24 h. Inhibition zones around the discs were measured in millimeters (mm). Positive controls included ampicillin (10 μg), chloramphenicol (30 μg), gentamicin (10 μg), erythromycin (15 μg) and amphotericin (10 μg).

### 2.9 Microencapsulation process

The encapsulation of *R. ribes* extract was conducted using maltodextrin (MD) and/or gum Arabic (GA) as carrier agents. Maltodextrin and gum Arabic can be appropriate candidate for encapsulating Rivas extract due to their safety, biocompatibility and good functional properties Therefore, the Rhubarb extract powder (15 % w/w of the carrier agent) and the wall material (20 % w/v in different ratios of MD:GA: 0:100 (MD); 50:50 (GA + MD); 0:100 (GA)) were combined with distilled water and mixed in an Ultra-Turrax homogenizer (KND-1200, UH1-013, South Korea) at 6000 rpm for 5 min, followed by stirring for 1 h at room temperature in the dark. The feed solutions were atomized into a lab-scale spray dryer (Dorsa Tech Co., Iran) using compressed air under the following experimental conditions: a feed rate of 1.5 mL/min, system pressure of 255 mbar, inlet temperature of 140 °C and airflow rate of 35 m^3^/h. The processing conditions were selected based on previous experience and literature carried out with relatively similar samples [25,[37], [38], [39]].

### 2.10 Encapsulation efficiency (EE)

Encapsulation efficiency refers to the percentage of phenolic compounds successfully entrapped into microparticles. To evaluate the surface phenolic content (SPC), 10 mg of encapsulated Rivas extract was dispersed in 10 mL of 96 % ethanol. The mixture was then centrifuged at 3000 rpm for 3 min and the phenolic content of the obtained supernatants was determined as a SPC [40]. EE% was calculated using the following equation (Eq. (2)):

(Eq. 2) $$\text{EE}\;\left(\%\right)=\frac{\;\text{TPC}-\text{SPC}}{\text{TPC}}\times 100$$

### 2.11 Morphology and particle size of encapsulated rhubarb extract

The morphology of rhubarb microparticles was examined using scanning electron microscopy (SEM: Tesca-Vega3, Tescan Co., Czech Republic) at an accelerating voltage of 10 kV [41]. The distribution and mean particle size of encapsulated Rivas extract were determined by measuring the diameter of 60 particles using Digimizer software (version 6.0; MedCalc Software, Belgium).

### 2.12 Moisture content

The moisture content of the different powders was assessed by drying samples in a vacuum oven (at 105 °C) until a constant weight was achieved and then expressed as a percentage [42].

### 2.13 Hygroscopicity

One gram of each sample was placed in a desiccator with a sodium chloride saturated solution (75.29 % relative humidity; at 25 °C). After 7 days, the weight of the samples and absorbed moisture was measured [43].

### 2.14 Solubility index (SI)

One gram of each microparticle (M1) was mixed in 25 mL distilled water and stirred for 30 min. This solution was then centrifuged at 3000 rpm for 10 min at 25 °C. The supernatants were dried at 105 °C and weighed (M2). The solubility value was calculated based on equation (3) [44].

(Eq. 3) $$\text{SI}\;\left(\%\right)=\frac{M2}{M1}\times 100$$

### 2.15 Bulk density

The bulk density of the samples was evaluated by dividing the mass of the powder by the volume it occupied in a 10 mL graduated measuring cylinder [45].

### 2.16 Color parameters

The color of different encapsulated samples was assessed using Hunter color parameters (L∗: degree of lightness on a scale of 0–100 from black to white; *a∗*: degree of redness (+) to greenness (−) and *b∗*: degree of yellowness (+) to blueness (−)) by a colorimeter (HunterLab Mini Scan EZ, USA) [39].

### 2.17 Statistical analysis

The ANOVA General Linear Model (GLM) was utilized for statistical analysis. Significant differences between the means were evaluated by the Tukey-Honest test (95 % confidence interval; P ≤ 0.05). Experiments were conducted in triplicate. Principal Component Analysis (PCA) examined the relationship between the variables assessed for microencapsulated Rivas flower extract. Minitab software (version 20, Minitab Inc. Pennsylvania, USA) was used for all analyses.

## 3 Results and discussions

### 3.1 Chemical compounds

**Fatty acids:** Fatty acids are the main chemical components of the Rheum genus essential oil [46]. The major saturated and unsaturated fatty acids in the *R*. *ribes* L. flower extract were palmitic-acid (3.66 %) and linoleic-acid (57.58 %). The extract mainly consists of unsaturated fatty acids, specifically linoleic-, oleic- and linolenic-acid (Table 1). Overall, the extract contained 8.83 % saturated fatty acids and 91.17 % unsaturated fatty acids. Therefore, Rivas flower extract obtained by the SCO_2_-EOH method was a rich source of unsaturated fatty acids.

**Table 1:**
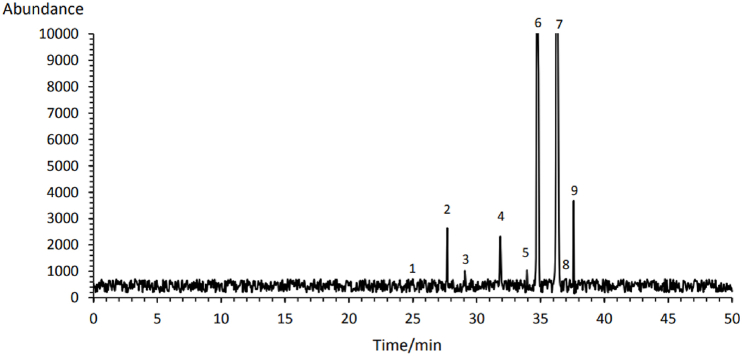
The profiles of free fatty acids of *Rheum ribes* Flowers extract obtained by supercritical CO_2_ and ethanol as cosolvent.

**Peak #**	**Chemical**	**RT (min)**	**Concentration (%w/w)**
1	Myristic Acid (C14:0)	25.02	0.99
2	Palmitic Acid (C16:0)	27.72	3.66
3	Palmitoleic Acid (C16:1)	29.08	1.41
4	Stearic Acid (C18:0)	31.86	3.20
5	Trans- Oleic Acid (t9C18:1+ t11C18:1)	33.94	1.44
6	**Cis- Oleic Acid (c9C18:1 + c6C18:1)**	**34.75**	**25.67**
7	**Linoleic acid (C18:2)**	**36.32**	**57.58**
8	Arachidic acid (C20:0)	37.02	0.98
9	Cis- Linolenic acid (c6,c9,c12C18:3)	37.63	5.07

RT: retention time.

Comparing these results with previous studies shows that the SCO_2_-EOH method was more effective in extracting unsaturated fatty acids than conventional methods. It was discovered that the essential oil of R. ribes stems contains 20.89 % total saturated fatty acids (with palmitic-acid at 15.22 % and stearic-acid at 5.67 %) and 79.11 % total unsaturated fatty acids (including palmitoleic-acid at 10.17 %, oleic-acid at 10.29 %, linoleic-acid at 20.40 %, linolenic-acid at 29.24 % and eicosapentaenioc-acid at 9.01 %) [47]. 9-Octadecenoic acid (ω-9) (42.8 %), 9, 12-Octadecadienoic acid (linoleic acid or ω-6) (19.6 %), hexadecanoic acid, (palmitic acid) (8.6 %), 1,2-benzenedicarboxylic acid diisooctyl (5.7 %), dodecane (3.7 %) and γ-linolenic acid (3.6 %) were identified as the fatty acids profile for *R. ribes* extracted by Dichloromethane [3]. However, Naemi et al. (2014) detected two fatty acids (palmitic acid: 27.08 % EO and linoleic acid: 6.56 % EO) and three fatty acid esters (Ethyl hexadecanoate: 1.30 % EO; 9,12,15-Octadecatrienoic acid methyl ester: 4.01 % EO and Ethyl linoleate: 4.76 % EO) in the GC-MS analysis of *R. ribes* essential oil flower [46]. Several factors, including plant parts, plant genotype, geographical location and climatic conditions may contribute to variations in the reported findings of the chemical composition of Rivas extract [[47], [48], [49]].

**Phytosterols:** The phytosterols found in Rivas flower extract were as follows: β-Sitosterol (197.02 mg/100g), Campesterol (144.47 mg/100g), Δ7-Avenasterol (73.59 mg/100g), Stigmasterol (13.05 mg/100g) and Brassicasterol (12.59 mg/100g) (Table 2). β-Sitosterol was also identified as the dominant phytosterol in *R. ribes* extract obtained by dichloromethane [3]. However, Keser et al. (2020) detected Stigmasterol (92.60 mg/g) and Ergosterol (31.45 mg/g) in the stem extract of Rivas. This variation could be due to differences in extraction methods, plant parts used, genotype, and climate conditions.

**Table 2:**
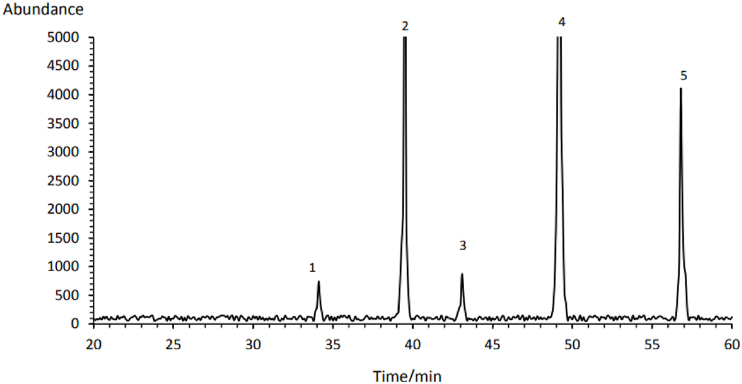
Phytosterol of *Rheum ribes* Flowers extract obtained by supercritical CO_2_ and ethanol as cosolvent.

**Peak #**	**Chemical**	**RT (min)**	**Concentration (mg/100g)**
1	Brassicasterol	34.10	12.56
**2**	**Campesterol**	**39.52**	**144.47**
3	Stigmasterol	43.09	13.05
**4**	**β-Sitosterol**	**49.21**	**197.02**
5	Δ7-Avenasterol	56.83	73.59

RT: retention time.

**Terpenoids:** Based on the results of GC-MS analysis, 29 terpenoid components were identified in the flower extract of Rivas (Table 3). The main terpenoid compounds in this extract were camphor (17.52 %), 1,8 cineol (10.91 %), borneol (7.76 %), camphene (7.47 %), α-pinene (6.91 %) and caryophyllene (5.66 %). Ragasa et al. (2017) identified α-pinene (13.5 %), terpinolene (12.4 %), *p*-cymene (10.6 %), bicyclogermacrene (9.6 %) and limonene (8.6 %) as the primary terpenoid components of *R. ribes* essential oil [3]. Additionally, germacrene D (22.3 %), α-pinene (13.5 %), terpinolene (12.4 %), *p*-cymene (10.6 %), bicyclogermacrene (9.6 %), limonene (8.6 %) and γ-Terpinene (0.6 %) were identified in Rivas essential oil [50]. Hasan and Köroğlu (2023) discovered that carvacrol (40.41 %) and gamma-terpinene (22.90 %) were the major terpenoid components of the *R. ribes* root extract. In contrast, the stem extract contained 2,4-ditert-butylphenol (20.76 %) and carvacrol (13.52 %), while the leaf extract had 2,4-ditert-butylphenol (25.87 %) and methyl formate (6.87 %) [51]. These discrepancies in results may be attributed to the differences in samples and extraction methods [49].

**Table 3:**
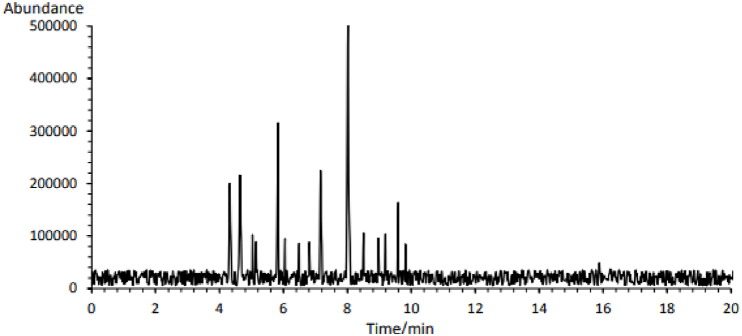
The terpenoids of *Rheum ribes* Flowers extract obtained by supercritical CO_2_ and ethanol as cosolvent.

**Peak #**	**Component**	**RT (min)**	**Concentration (%)**	**Peak #**	**Component**	**RT (min)**	**Concentration (%)**
**1**	**α-pinene**	**4.32**	**6.91**	16	Acetic acid	9.04	0.99
**2**	**Camphene**	**4.64**	**7.47**	17	T-β terpinyl butanoate	9.19	3.58
3	pinene	5.03	3.52	18	**Caryophyllene**	**9.59**	**5.66**
4	β-myrcene	5.14	3.06	19	linalool	9.82	2.92
5	Sabinene	5.23	0.31	20	α-caryophyllene	10.11	1.09
**6**	**1,8 Cineol**	**5.83**	**10.91**	21	Copaene	10.87	0.56
7	β-phellandrene	5.91	1.19	22	Menthol	12.03	0.86
8	α-terpineol	6.05	3.27	23	Cyclohexane,1,1,2-trimethy	13.01	1.09
9	D-limonene	6.49	2.96	24	(+)-globulol	13.39	0.86
10	Thujone	6.81	3.04	25	Epiglobulol	13.68	1.19
**11**	**Borneol**	**7.16**	**7.76**	26	Methy(z) 8,11,14 Eicosatrienoate	14.03	1.05
12	P-menth-1-en-8-ol	7.35	1.12	27	Humulane-1,2-dien-3-ol	15.41	1.15
**13**	**Camphor**	**8.02**	**17.52**	28	Oxaspiro(2,5)octane,5,5 dimethyl	15.88	1.68
14	P-cymen	8.51	3.65	29	1-Naphthalene propano	16.23	1.27
15	P-menth-1-en-β-ol	8.97	3.30				

RT: retention time.

**Phenolic Compounds:** The composition of flavonoids and phenolic acids in *R. ribes* flowers is detailed in Table 4. The major phenolic compounds in the flower extract were m-Coumaric acid (48.22 mg/g), luteolin (38.07 mg/g), gallic-acid (26.25 mg/g), quercetin (22.50 mg/g), chlorogenic acid (21.42 mg/g), kaempferol (16.50 mg/kg), caffeic acid (14.95 mg/g), rutin (13.99 mg/g) and quercitrin (9.68 mg/g) (Table 4). In a study by Keser et al. (2020), caffeic acid (302.45 mg/g), ferulic acid (269.25 mg/g), rutin (15.90 mg/g) and catechin (24.85 mg/g) were identified as major phenolic compounds in ethanol extracts of *R. ribes* stems [47].

**Table 4:**
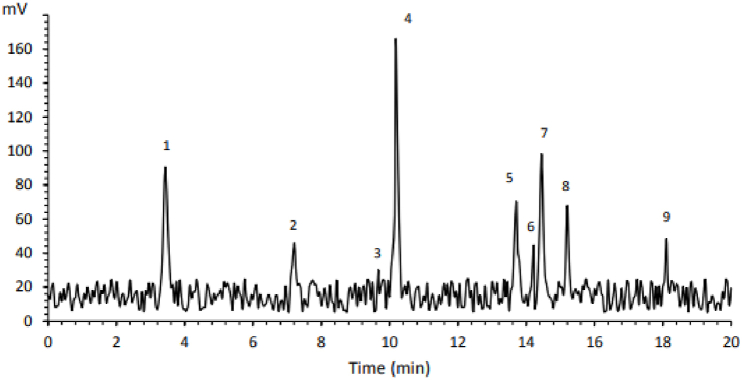
The profiles of phenolic compounds of *Rheum ribes* Flowers extract obtained by supercritical CO_2_ and ethanol as cosolvent.

**Peak #**	**Chemical**	**RT (min)**	**Concentration (mg/g)**
1	Gallic acid	3.44	26.25
2	Rutin	7.21	13.99
3	Quercitrin	9.67	9.68
**4**	**m-Coumaric acid**	**10.18**	**48.22**
5	Quercetin	13.71	22.50
6	Caffeic acid	14.22	14.95
7	Luteolin	14.45	38.07
8	Chlorogenic acid	15.22	21.42
9	Kaempferol	18.11	16.50

RT: retention time.

Emodin, aloe-emodin, physcion, chrysophanol, rhein, chlorogenic acid, rutin, gallic acid, tannic acid, and kaempferol were found in *R. ribes* ethanol and aqueous root extracts [52]. Despite the enhancement of CO_2_ polarity with a small amount of ethanol, the higher level and diversity of phenolic compounds reported in previous studies may be attributed to the polar nature of phenolic compounds and their greater tendency to polar solvents [7]. Furthermore, the chemical composition of an extract can be influenced by the plant part and location. For example, phenolic compounds of *R. ribes* roots included gallic acid, ellagic acid, quercetin, catechin, rutin, cinnamic acid, tannic acid, emodin, aloe-emodin, and physcion. Cinnamic acid and tannic acid were not observed in the extracts of leaves and flowering stems of Rivas [53]. Overall, the investigation into the chemical composition of Rivas extract obtained using SCO_2_-EOH showed that this extract contains a greater variety of bioactive compounds compared to extracts obtained using conventional methods based on polar or non-polar solvents. This suggests that the SCO2-EOH extraction method can result in extracts with higher bioactivity.

### 3.2 Total phenolic (TPC) and flavonoid content (TFC)

The TPC and TFC of *R. ribes* flower extract were measured at 306.19 ± 13.59 mg GAE/g and 179.84 ± 5.77 mg QE/g respectively. Previous studies have indicated that the flowering parts of *R. ribes* contain high concentrations of phenolics, flavonoids, terpenoids, and glycosides [49,54]. Despite the modification of CO_2_ polarity by the addition of ethanol, the phenolic compounds extracted by SCO_2_-EOH were lower than those reported for *R. ribes* extracts obtained using polar solvents [47,53,55].

### 3.3 Antioxidant activity

The DPPH radical scavenging activity of the Rivas extract was 89.6 ± 1.39 %, which is consistent with previous reports. Keser et al. (2020) found that the water, ethanol and methanol extracts of *R. ribes* stem exhibited radical scavenging ratios of 93.49 %, 94.21 % and 95.86 %, respectively [47]. The water and methanol extracts of *R. ribes* stem showed DPPH radical scavenging activities of 83.90 % and 87.07 % respectively [56,57]. The methanol extract of *R. ribes* demonstrated a DPPH radical scavenging potential of 84.5 % [4].

Moreover, the current study results revealed that there were no significant differences between the antioxidant activity of the *R. ribes* flower extract and 100 ppm of BHT, a common synthetic antioxidant (90.11 ± 0.89 %). The strong antioxidant potential of the *R. ribes* flower extract may be attributed to various bioactive compounds, such as phenolic compounds, carotenoids, phytosterols, terpenes and tocopherols extracted using the SCO_2_-EOH method [6,7,58].

### 3.4 Antimicrobial potential

The R. ribes flower extract demonstrated effective zones of inhibition in a dose-dependent manner against gram-negative (*E. coli*), gram-positive (L. monocytogenes) bacteria, and mold (A. fumigatus) (Table 5). As the concentration of the extract increased, the zones of inhibition for *E. coli*, L. monocytogenes, and A. fumigatus also increased (P < 0.05). The inhibition zone for gram-positive bacteria and A. fumigatus was higher than that observed for *E. coli* as a gram-negative bacterium. Previous reports have indicated that Gram-negative bacteria were less susceptible to extracts from Rheum rhaponticum L. roots and petioles compared to Gram-positive bacteria [59]. The lipopolysaccharide membrane of gram-negative bacteria can protect the cytoplasmic membrane from antimicrobial compounds [60,61]. However, it has been reported that bioactive compounds can be embedded in the lipid layer of the cell membrane of gram-negative bacteria and interacts with the polar part of the membrane through hydroxyl groups and with the hydrocarbon tail of the membrane via the hydrophobic part. The outcome of this process is the destabilization of the lipid bilayer of the outer membrane which causes leakage of cytoplasmic ions, change in the proton motive force of the bacterial cell and ultimately leads to the lysis of the cell's contents [5].

**Table 5:** Antimicrobial activity of *R. ribes* flowers extract obtained by SCO_2_-EOH against foodborne pathogen bacteria by disc diffusion method.

Treatment	Concertation	Disc-diffusion method (mm)		
		*E. coli*	L. monocytogenes	A. fumigatus
*R. ribes* flowers extract	A: 50 μg/Ml			
	B: 100 μg/mL			
	C: 200 μg/mL			
	D: 400 μg/mL			
	50 μg/mL	R	**R**	**R**
	100 μg/mL	R	8.10 ± 0.31 ^E^	7.40 ± 0.55 ^B^
	200 μg/mL	8.67 ± 0.76 ^C^	12.00 ± 0.70^D^	8.20 ± 0.62 ^B^
	400 μg/mL	14.00 ± 0.50 ^B^	15.13 ± 0.61 ^C^	12.30 ± 0.79^A^
Ampicillin a	10 μg/mL	7.43 ± 0.40^D^	27.07 ± 0.90^A^	R
Chloramphenicol^a^	30 μg/mL	16.67 ± 0.57^A^	23.97 ± 0.90 ^B^	R
Gentamicin a	10 μg/mL	9.23 ± 0.25 ^C^	12.87 ± 0.32^D^	R
Erythromycin	15 μg/mL	R	22.66 ± 1.04 ^B^	R
Amphotericin	10 μg/mL	R	–	8.13 ± 0.65 ^B^

R: resistant, data represents as mean ± SD (n = 3). Different superscripts in the same column correspond to a significant difference between treatments (P *<* 0.05).

a Positive control antimicrobial agents.

The extract at 50 μg/mL showed no antimicrobial effect. There was no visible antibacterial activity against *E. coli* at 100 μg/mL. When comparing the antibacterial effects of the rhubarb flower extract with synthetic antibiotics on *E. coli*, it was found that at 200 μg/mL, the extract had a higher potential for antibacterial effect than Ampicillin (P < 0.05) and similar effects to Gentamicin (P > 0.05). At 400 μg/mL, the extract's antibacterial activity was significantly higher than the control positive samples except for Chloramphenicol (P < 0.05). Erythromycin at 15 μg/mL did not show any visible antibacterial effect against *E. coli*. Overall, the antibacterial performance of the extract (at a concentration of >200 μg/mL) against *E. coli* was comparable to that of conventional synthetic antibiotics (Table 5).

The antibacterial effect of this extract at 100 μg/mL against *L. monocytogenes* is significantly lower than that of Ampicillin, Chloramphenicol and Erythromycin. However, at a concentration of 200 μg/mL, there was no significant difference between the inhibition zones formed by the extract and Erythromycin. At 400 μg/mL, the antibacterial effect of the extract was significantly higher than Gentamicin (P < 0.05). Therefore, it seems that a concentration of approximately 400 μg/mL of this extract is suitable to achieve an antibacterial effect comparable to synthetic antibacterial agents against *L. monocytogenes* and likely other Gram-positive bacteria.

The anti-fungal effect of *R. ribes* flower extract (at 100 and 200 μg/mL) against *A. fumigatus* was significantly similar to that of Amphotericin. The effectiveness of this extract at 400 μg/mL was significantly higher than that of Amphotericin. Therefore, the extract had a suitable antifungal effect against *A. fumigatus* even at relatively low concentrations compared to Amphotericin.

Research has indicated that terpenoids reduce the ergosterol concentration in the cell membrane of molds, elevate the concentration of reactive oxygen species, and induce oxidative stress. These processes subsequently decrease the amount of extracellular polymeric matrix, a type of capsular polysaccharide, and disrupt the integrity of the cell membrane [5].

Keser et al. (2020) also reported the antimicrobial activity of *R. ribes* methanol against *E. coli* and *L. monocytogenes*. The antibacterial effects of rhubarb may be related to various bioactive compounds such as phenols, flavonoids, unsaturated fatty acids, terpenoids, enzymes, etc., extracted by SCO_2_-EOH [1,50,52,62].

It was reported that unsaturated fatty acids can exhibit antimicrobial activity against *S. aureus, P. aeruginosa, B. subtilis, C. albicans,* and *T. mentagrophytes* [3]. In general, rhubarb extract obtained by the SCO_2_-EOH extraction method can be a promising alternative to synthetic antimicrobial agents in food.

### 3.5 The physicochemical properties of encapsulated extract

#### 3.5.1 Encapsulated efficiency (EE %)

The EE % of R. ribes microparticles is presented in Table 6. Encapsulation efficiency values ranged from 90.53 % (MD) to 93.23 % (GA + MD) (P < 0.05). However, the difference between GA (91.37 %) and the other samples was not significant. Araujo et al. (2022) also found that the EE% of spent coffee grounds extract encapsulated in GA and GA + MD was higher than that encapsulated in MD. The superior EE% of microparticles based on gum Arabic can be attributed to its structure. The branching structure and good emulsifying properties of GA along with the presence of a small amount of protein in GA structure attached to carbohydrate chains (via covalent bonds) make GA a suitable candidate for film formation and trapping bioactive compounds. The lower EE% of MD is likely due to its lower emulsifying ability and film-forming capacity [29]. Overall, the difference in EE% among various samples may be due to interactions between phenolic compounds and carrier agent compositions. The encapsulation efficiency is affected by the type and level of the carrier and core materials as well as medium conditions, especially pH [[63], [64], [65]]. The EE% of Rivas extract microparticles was similar to those reported for various microparticles [21,66,67].

**Table 6:** Encapsulation efficiency and physical properties of microencapsulated powders loaded with Rivas flower (*Rheum* ribes) extract.

	MD	GA	GA + MD
Mean particle size (μm)	34.48 ± 27.56^B^	46.70 ± 28.20^A^	45.30 ± 28.60^A^
Encapsulation efficiency (%)	90.53 ± 1.14^B^	91.37 ± 0.88^AB^	93.24 ± 1.10^A^
Moisture content (%)	3.32 ± 0.11^B^	4.18 ± 0.09^A^	2.81 ± 0.22^C^
Hygroscopicity (%)	21.23 ± 0.60^A^	19.13 ± 0.50^B^	20.53 ± 0.47^A^
Bulk density (g/cm^3^)	0.43 ± 0.02^A^	0.40 ± 0.01^A^	0.41 ± 0.1^A^
Solubility index (%)	96.17 ± 0.47^A^	93.07 ± 0.35^C^	94.37 ± 0.61^B^
L∗	66.33 ± 1.26^B^	71.83 ± 0.76^A^	72.67 ± 1.53^A^
a∗	30.67 ± 0.58^C^	35.33 ± 1.15^B^	41.50 ± 0.87^A^
b∗	−0.47 ± 0.50^B^	1.83 ± 0.76^A^	2.53 ± 0.51^A^

Data represents as mean ± SD (n = 3). Different superscripts in the same row correspond to a significant difference between various microparticles (P *<* 0.05); MD: Rivas flower extract encapsulated in maltodextrin; GA: Rivas flower extract encapsulated in gum Arabic; GA + MD: Rivas flower extract encapsulated in gum Arabic and maltodextrin (50:50).

#### 3.5.2 Morphology and particle size

**Fig. 2:**
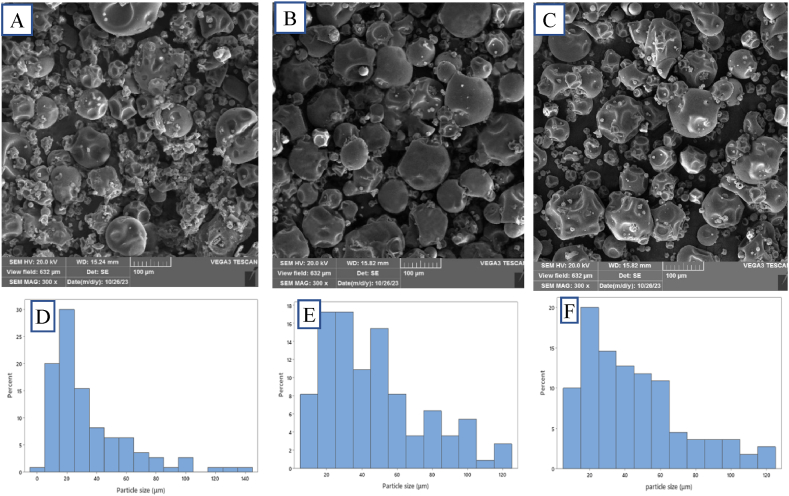
Morphology of the encapsulated *Rheum ribes* extract encapsulated in (A) MD, (B) GA, (C) GA + MD (50:50); and particle size distribution of (D) MD particles, (E) GA particles and (F) GA + MD particles; MD: Maltodextrin; GA: Gum Arabic.

Spray-dried particles exhibited irregular spherical and semi-spherical shapes with wrinkled surfaces (Fig. 2). Moreover, there were almost no visible cracks on the surfaces of the samples. Surface irregularities may enhance the dispersibility and rehydration of the powder [39]. A comparison of GA and/or MD particles revealed that the wrinkles on the surface of the MD particles were more pronounced than those on the GA particles (Fig. 2A–B). This difference could be attributed to the faster moisture removal and rapid cooling in the MD samples [22]. Smoother particles tend to be more stable, resulting in a more controlled release rate of core materials [25].

A slight agglomeration was observed in the MD sample. GA + MD microparticles exhibited smaller and more spherical shapes with a smoother surface compared to MD ones (Fig. 2C). In this regard, Pudziuvelyte et al. (2019) also reported a similar behavior in microparticles containing *Elsholtzia ciliata* ethanolic extract encapsulated with a combination wall material compared to particles produced only by one coating agent [68]. The water evaporation rate, particle size, the type and concentration of wall material, wall-to-core ratio, and chemical composition of core agents, can be some of the reasons that affect the structure of spray-dried microparticles [69].

As shown in Fig. 2D–F, the particle size of all samples was not uniform. The microparticles ranged in size from 0.5 to 142 μm. The average particle size of MD (34.48 ± 27.56 μm) was significantly lower than GA + MD (45.30 ± 28.60 μm) and GA (46.70 ± 28.20 μm) (Table 6). This difference may be attributed to the better reconstitution properties of MD particles (see section 3.5.3). The higher particle size of GA may be due to its higher viscosity compared to MD [22]. Furthermore, larger particles may offer better protection for the core material against oxidation, while smaller particles with a larger surface area may lead to degradation of bioactive compounds [21,25].

There was no significant difference between the particle size of GA + MD and GA. Previous reports have shown a wide range of particle sizes (1 nm to over 150 μm) for spray-dried powders [25,28,40,[70], [71], [72], [73]]. This variation could be due to factors such as inlet air temperatures, spray dryer nozzle size, viscosity of the feed solvent, type of wall, and core materials [27,28].

The particle size distribution for MD was around 50 % for particles <20 μm, while for the GA sample, the most frequent size (⁓52 %) was for particles between 25 and 65 μm. The highest frequency size (59 %) for GA + MD powders was 15–55 μm (Fig. 2D–F). Therefore, the combination of GA and MD has resulted in the production of a powder with a more uniform distribution.

#### 3.5.3 Basic physical properties

**Moisture Content:** The moisture content of powders is closely linked to various factors such as water activity, drying efficiency, stability, flowability, stickiness and shelf-life (including microorganism growth and oxidation). High moisture levels can lead to a transition from a glassy state to an amorphous rubbery state in wall materials, resulting in degradation and release of the core material [39]. On the other hand, low moisture content reduces the water's ability to act as a plasticizer and decreases the glass transition temperature [74]. Maintaining a moisture content of around 2–5% can enhance the shelf-life of powder products [70].

According to Table 6, GA particles had a higher moisture content (4.18 %) compared to MD (3.32 %) and GA + MD (2.81 %). The difference between the samples was statistically significant (P < 0.05).

The higher moisture content of the GA sample may be attributed to the greater number of hydrophilic groups in gum-Arabic compared to starch derivatives such as MD, which can easily bond to water molecules and retain moisture [67,75]. These results were consistent with findings from various encapsulated extracts such as pink pepper/green propolis (2.52–3.33 %) [67], jamun juice (2.57–3.31 %) [73], eggplant peel (2.34–4.11 %) [75] and blackberry (3.25–4.43 %) [25]. However, they were lower than the moisture content found in microparticles containing fingered citron extract (4.50–5.70 %) [39]. The variations in findings may be attributed to differences in spray-drying conditions, types and levels of microparticle agents (wall and core material) [25,75].

**Hygroscopicity:** The ability of materials to absorb moisture from the surrounding environment is known as hygroscopicity, impacted the shelf-life, stability and fluidity of powders [67]. The lowest and highest hygroscopicity values were recorded for MA (21.24 %) and GA (19.13 %) respectively (Table 6). The difference between the GA sample and the others was significant (P < 0.05). The hygroscopicity of spray-dried powders is influenced by the type and concentration of encapsulation agents as well as particle size affect [76]. Laureanti et al. (2023) also observed lower hygroscopicity in microcapsules produced with GA + MD compared to those produced with only MD [67]. This difference may be attributed to the higher glass transition temperature of GA compared to MD [75]. The presence of more hydrophilic groups and smaller particle size in MD results in faster moisture absorption from the environment [73]. Similar findings have been reported for pink/green pepper (11.6–16.4 %) [67], eggplant peel (14.91–20.72 %) [75] and Jamun juice (21.52–25.86 %) [73] particles.

**Bulk Density (BD):** BD is one of the most crucial physical properties of particles that can impact the transportation, storage, and packaging of a product [77]. The bulk density values of the particles ranged from 0.43 to 0.40 g/cm^3^ (P > 0.05; Table 6). The bulk density of powders can be influenced by the molecular weight of the carrier agent [73]. Particles with higher moisture content tend to agglomerate, reducing the space between them and consequently increasing bulk density [29,75]. Similar results have been reported by previous authors for various extracts encapsulated in MD and/or GA using spray-drying [26,39,73], but higher than those obtained for chipilin leaf extract microparticles (0.210–0.260 g/cm^3^) [71]. A higher bulk density indicates a lower amount of air in the powder, which can help delay lipid oxidation during storage [39].

**Solubility Index:** The good solubility of powders makes it easier to use microparticles in food formulation [75]. A significant difference was observed in the solubility values of *R. ribes* extract microparticles (Table 6). The solubility values ranged from 93.07 % (GA) to 96.17 % (MD) (P < 0.05). These values are similar to those reported for various extract encapsulated in MD and/or GA such as Moringa stenopetala leaf extract particles (80.52–92.92 %), eggplant peel extract microcapsules (94.31–98.78 %) [75] and roselle powder (94.1–98.5 %) [40]. Maltodextrin has high solubility, making it a suitable material for the drying process [71].

**Color Parameters:** The color parameters (*L∗*, *a∗* and *b∗*) are presented in Table 6. There was a significant difference between the color parameters of various samples (P < 0.05). Lightness values (*L∗*) ranged from 66.33 (MD) to 72.67 (GA + MD). The difference between the samples was significant at a 5 % level. Mean values of *a∗* ranged from 30.67 (MD) to 41.50 (GA + MD) (P < 0.05). Therefore, samples tend to be of a red color. Mean values of *b∗* ranged from −0.47 (MD) to 2.53 (GA + MD) indicating MD samples tend to be of a blue color and the other two samples had a yellow tone. The wall material also had a significant effect on the *b∗* mean value. The color was influenced by the spray-drying condition, type and level of wall and core materials [22]. Color differences between samples can be related to the inherent color of wall materials and *R. ribes* extract [25,28,71,75].

### 3.6 Principal component analysis (PCA)

PCA can illustrate the relationship between various primary variables (price, MC: moisture content, Hyg: Hygroscopicity, SI: solubility index, BD: bulk density, EE: encapsulation efficiency, mean particle, *L∗*: Lightness) using new variables (i.e., principal components: PC). Since maltodextrin has a lower price than gum Arabic (0.9–1.5 $ vs 2.7–4.5 $), a price score of 2 was assigned to MD. Scores of 1 and 1.5 were given to GA and GA + MD, respectively.

**Fig. 3:**
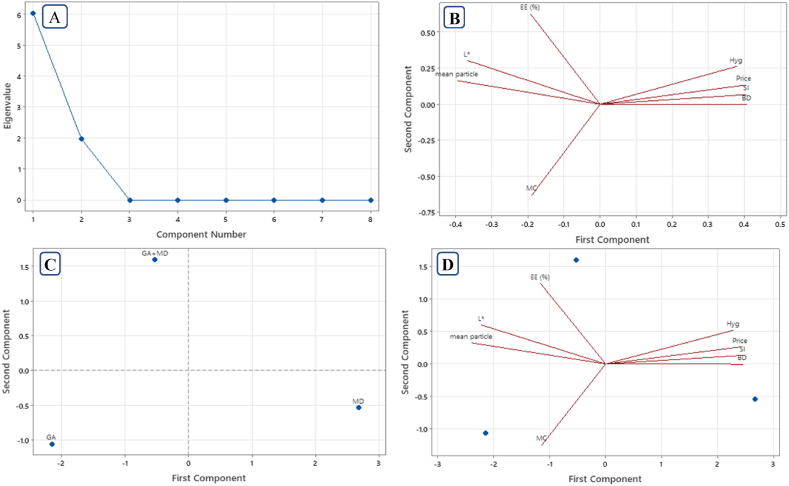
A) Scree plot; B) Correlation loading plot; C) Score plot and D) biplot (correlation loading plot and score plot) from principal components analysis (PCA) of powders loaded with Rivas flower (*Rheum* ribes) extract and encapsulated by maltodextrin (MD), gum Arabic (GA) and maltodextrin-gum Arabic (GA + MD). BD: bulk density (g/cm^3^); EE: Encapsulation efficiency (%); Hyg: Hygroscopicity (%); L: lightness; MC: moisture content (%); mean particle: Mean particle size (μm); SI: solubility index (%).

Fig. 3 displays the results of the PCA analysis. The plot of eigenvalues vs. PC numbers or scree plot (Fig. 3 A) indicates that the first two principal components retained 100 % of the total variance. Principal Component-1 (PC_1_) accounted for 75.3 % and Principal Component-2 (PC_2_) explained 24.7 % of the total data variance. Each principal component can help identify the properties of microparticles. By examining the loadings (Fig. 3B), it is evident that the key variables explaining the first principal component (PC_1_) were BD (0.407), SI (0.405), price (0.400), mean particle (−0.396), Hyg (0.379) and *L∗* (−0.369), respectively (Fig. 3 B). PC_2_ was characterized by moisture content (−0.631) and encapsulation efficiency (0.627). Since there was a positive correlation between EE and the stability of bioactive compounds [21,23], PC_2_ reflects the sample stability.

The most suitable sample was selected based on the direction of eigenvectors [78,79]. According to the score plot (Fig. 3C), the scores of the GA + MD sample are located on the negative side of PC_1_ and the positive side of PC_2_, while the scores of the GA samples were negative in both PC_1_ and PC_2_ and the MD sample was positioned on the positive side of PC_1_ and negative side of PC_2_. Therefore, the properties of the powders obtained from various wall materials differed. It has been reported that PCA can verify differences between samples [64,80].

Since the physicochemical properties of all obtained powders were acceptable, according to PCA, when considering physical features, especially solubility and price, the MD sample may be the best choice. Therefore, the MD sample could be a suitable and cost-effective option to enhance the nutritional value of food formulations.

If the stability of bioactive compounds is a concern, particularly when using powders as a natural preservative, the GA + MD sample demonstrates an advantage over others. Additionally, the GA + MD sample had higher lightness than the others. Despite the gum Arabic sample showing acceptable properties, the higher cost of GA compared to maltodextrin has prevented it from providing a competitive advantage over the other two samples.

## 4 Conclusions

This study demonstrated that the Rivas flower extract obtained by the SCO_2_-EOH method contains a wide variety of bioactive compounds, including unsaturated fatty acids, phytosterols, terpenoids and phenolic acid. The extract showed a relatively high levels of phenolic and flavonoid content, indicating its antioxidant potential. The DPPH radical scavenging activity of the Rivas extract was comparable to the antioxidant activity of BHT. This extract is suitable for achieving an antimicrobial effect comparable to synthetic antimicrobial agents. Therefore, this extract can be used as a natural preservative in the formulation of food products sensitive to microbial and chemical spoilage, such as meats, nuts, dairy products and etc. to prolong their shelf-life. It can be also used in the formulation of bioactive edible films/coatings. The resulting spray-dried particles had desirable characteristics such as acceptable color, low moisture content, and excellent solubility. According to the PCA, maltodextrin samples showed the best solubility and the lowest price, making them a cost-effective ingredient for improving the nutritional value and formulating various functional and healthy foods such as beverages and desserts. If the stability of bioactive compounds are more important features, MD + GA sample will be the best choice. Although the gum-Arabic sample also showed suitable physicochemical properties, it did not provide a competitive advantage over the other samples. Further research is needed to assess the chemical structure, release kinetics, thermodynamic behavior of these microparticles as well as performance of these spray-dried powders in food formulations.

## CRediT authorship contribution statement

**Seyyed Ali Hoseini:** Writing – original draft, Software, Methodology, Investigation, Formal analysis, Data curation. **Mohsen Vazifedoost:** Writing – original draft, Visualization, Validation, Supervision. **Bahareh Hajirostamloo:** Validation, Supervision. **Zohreh Didar:** Validation, Supervision. **Mohamad Mehdi Nematshahi:** Validation, Supervision, Software, Formal analysis.

## Data availability

The datasets for the current study are available from the corresponding author upon reasonable request.

## Ethical approval

This article does not involve any studies with animals conducted by any of the authors.

## Funding

This study was conducted at the authors’ personal expense and recieved no sponsorship.

## Declaration of competing interest

The authors declare that they have no conflict of interest.
